# Fluoroscopy-Assisted C1–C2 Posterior Fixation for Atlantoaxial Instability: A Single-Center Case Series of 78 Patients

**DOI:** 10.3390/medicina58010114

**Published:** 2022-01-12

**Authors:** Charles Tatter, Alexander Fletcher-Sandersjöö, Oscar Persson, Gustav Burström, Erik Edström, Adrian Elmi-Terander

**Affiliations:** 1 Department of Neurosurgery, Karolinska University Hospital, 171 64 Stockholm, Sweden; alexander.fletcher-sandersjoo@ki.se (A.F.-S.); oscar.persson.1@ki.se (O.P.); gustav.burstrom@ki.se (G.B.); erik.edstrom.1@ki.se (E.E.); adrian.elmi.terander@ki.se (A.E.-T.); 2 Department of Clinical Neuroscience, Karolinska Institutet, 171 77 Stockholm, Sweden

**Keywords:** atlantoaxial instability, C1–C2 posterior fixation, non-navigated surgery, fluoroscopy-assistance, case series

## Abstract

*Background and Objectives*: Posterior C1–C2 fixation, with trans-articular screws (TAS) or screw-rod-construct (SRC), is the main surgical technique for atlantoaxial instability, and can be performed with a fluoroscopy-assisted free-handed technique or 3D navigation. This study aimed to evaluate complications, radiological and functional outcome in patients treated with a fluoroscopy-assisted technique. *Materials and Methods*: A single-center consecutive cohort study was conducted of all adult patients who underwent posterior C1–C2 fixation, using TAS or CRS, between 2005–2019. *Results*: Seventy-eight patients were included, with a median follow-up time of 6.8 years. Trauma was the most common injury mechanism (64%), and cervicalgia the predominant preoperative symptom (88%). TAS was used in 33%, and SRC in 67% of cases. Surgery was associated with a significant reduction in cervicalgia (from 88% to 26%, *p* < 0.001). The most common complications were vertebral artery injury (*n* = 2, 2.6%), and screw malposition (*n* = 5, 6.7%, of which 2 were TAS and 3 were SRC). No patients deteriorated in their functional status following surgery. *Conclusions*: Fluoroscopy-assisted C1–C2 fixation with TAS or SRC is a safe and effective treatment for atlantoaxial instability, with a low complication rate, few surgical revisions, and pain relief in the majority of the cases.

## 1. Introduction

Atlantoaxial instability can be caused by odontoid fractures, arthritis and cervical tumors [1,2,3,4]. If left untreated, the destabilized atlantoaxial segment may lead to excessive translational and rotational movements resulting in neck pain and the risk of spinal cord compression [5,6]. The atlantoaxial transarticular screw (TAS) technique, introduced by Magerl [7], and the screw and rod construct (SRC) technique, described by Goel and Harms [8,9], both provide atlantoaxial stability without the need for external immobilization [5,10,11]. However, the techniques also carry a risk of vertebral artery injury (VAI) or spinal cord injury if the screws are placed too laterally or medially, respectively [12]. During recent years, navigation systems and templates for assisted screw positioning have been introduced to reduce the frequency of screw misplacement. Despite the known advantages of navigated surgery [13], its implementation for spine surgery has been slower than for corresponding cranial applications [14] partly due to the relatively complicated and time-consuming setup of spinal navigation devices. As a result, many centers still use non-navigated, fluoroscopy-guided techniques for atlantoaxial fixations.

At the study center, a transition towards navigated C1–C2 fixation has recently taken place. In that context, the aim of this retrospective single center study was to evaluate and present our historical experience with non-navigated, fluoroscopy-assisted C1–C2 posterior fixation for atlantoaxial instability, paying special attention to safety and outcomes.

## 2. Materials and Methods

### 2.1. Patient Selection and Study Setting

The study is a retrospective, consecutive, case series. All adult patients (≥18 years) who underwent C1–C2 posterior fixation for atlantoaxial instability, with either TAS or SRC, at the study center between 2005–2019 were eligible for inclusion. Patients treated with hybrid techniques or constructs involving softwires for stability, as well as those without any follow-up imaging, were excluded. In total 78 patients were included, 26 operated with TAS, ad modum Magrl and 52 with SRC, ad modum Harms. The study hospital is a publicly funded and owned tertiary care center serving a region of roughly 2.3 million inhabitants, and the only neurosurgical center in the region. Patients were identified using the surgical management software Orbit (Evry Healthcare Systems, Solna, Sweden). Medical records and imaging data from digital hospital charts were retrospectively reviewed using the health record software TakeCare (CompuGroup Medical Sweden AB, Farsta, Sweden), and regional electronic archives. The Regional Ethical Review Board approved the study (Dnr: 2016/1708-31/4) who waived the need for informed consent. This case series has been reported in line with the PROCESS Guideline [15].

### 2.2. Transarticular Screw Technique

Using a midline incision, the facet joints of C2-C3 were exposed bilaterally with minimal muscle detachment. Two small transverse incisions (<1 cm) were made at approximately C5-C6 level depending on the required optimal angle decided by fluoroscopy. A percutaneous trocar was inserted, and the tip was placed at the base of C2-C3 facet joints. A guidewire was then drilled under fluoroscopic surveillance until the anterior arch of C1 was reached. A cannulated screw (3.5 mm in diameter) was then placed over the guidewire and the final position was confirmed with fluoroscopy. All incisions were sutured according to routine.

### 2.3. Screw and Rod Construct Technique

Using a midline incision, the arch of C1 and the cranial surface of the isthmus of C2 were exposed bilaterally with minimal muscle detachment. Polyaxial screws (Synapse, Synthes, Johnson & Johnson, Raynham, MA, USA), 3.5 mm in diameter, mostly 30 mm in length, were used. For C1 lateral mass screw insertion a custom-made drill and screw guide (Synthes, Johnson & Johnson, Raynham, MA, USA) was used. The dorsal root ganglion of C2 was retracted caudally. Bilateral drilling and placing of the screws were performed through the guide without removing it from the fluoroscopically verified position. C2 pedicle screws were then placed parallel to the C1 screws, as high in the pedicle as possible to avoid the vertebral artery. Either a drill or a gearshift was used to prepare the screw canal under fluoroscopic guidance. Integrity of the pilot hole was verified with a blunt probe. In case of high rising vertebral arteries, where pedicles screws were not deemed safe, lamina screws could instead be placed in C2 according to the surgeon’s discretion. Finally, rods were placed and fixed. The incision was sutured according to routine.

For both TAS and SRC, follow up with CT scan was performed at day 1, and at 4 and 12 weeks postoperatively, followed by a standardized clinical follow-up at the outpatient clinic. No collars were used.

### 2.4. Statistics

Shapiro-Wilk test was used to evaluate the normality of the data. All continuous data significantly deviated from a normal distribution pattern (Shapiro-Wilk test *p* value < 0.05) and is therefore presented as median (range) while categorical data is shown as numbers (proportion). McNemar’s test (paired nominal data) was used to determine whether there was a statistically significant improvement in neurological function following surgery. Statistical significance was set at *p* < 0.05. All analyses were conducted using the statistical software program R, utilizing the graphical interface RStudio^®®^ (RStudio, Boston, MA, USA) [16].

## 3. Results

### 3.1. Baseline Data

In total, 91 patients were assessed and 78 met the inclusion criteria. Thirteen patients were excluded, 12 due to the use of other surgical techniques than TAS or SRC and 1 due to lack of follow up CT-scan (only MRI). Median age at the time of surgery was 63 years, 46% of the patients were male. Three of the patients had previously undergone anterior dens screw fixation and 1 was previously treated with a C1–C2 fixation with softwire. Trauma was the most common surgical indication (*n* = 50, 64%), while 9 (12%) cases were treated for rheumatic instability, 3 (3.8%) cases for odontoid-related instability, 2 (2.6%) for pseudarthrosis despite previous surgery and the remaining 14 (18%) for other degenerative atlantoaxial disorders. The most common traumatic injury was isolated dens-type 2-fracture (*n* = 26). Twenty-seven of the trauma patients were treated with acute C1–C2 stabilization within the first week from the trauma, while the remaining 23 trauma patients were initially treated conservatively but later converted to surgical treatment due to increased instability (non-union) and cervicalgia. Pain (cervicalgia) was also the most prevalent clinical finding in the entire cohort (*n* = 69, 88%). The most common preoperative Frankel grade was E (*n* = 65, 83%) (normal motor and sensory function). There were no cases of complete spinal cord injury (Frankel grade A). Pre-operative MRI was performed in 46 (59%) cases, and 9 (12%) had intramedullary high signal intensity on MRI. Forty-three (55%) patients had a pre-operative CT-angiography (Table 1). Data comparing TAS and SRC groups regarding baseline, treatment and outcome are presented in Supplementary Table S1.

### 3.2. Treatment Data

**Figure 1:**
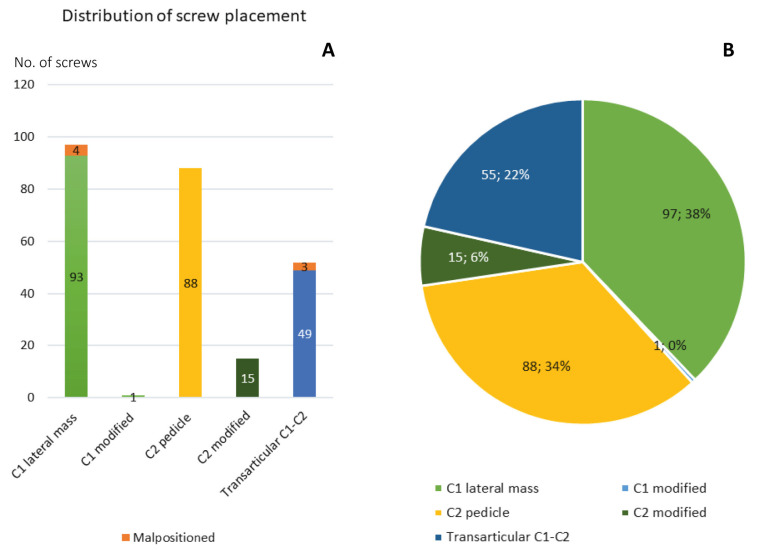
Distribution of screw placement. (**A**) Histogram of the distribution of screw placement types along with the number of malpositioned screws. (**B**) Relative distribution of screw placement types in absolute and numbers and percentages.

The median time between diagnosis and surgery was 84 (0–3327) days. For trauma patients, the median time to surgery was 2 (0–7) days. The median operation room (OR) time was 150 (64–306) min and the median perioperative blood loss 350 (25–2100) mL. The surgical method was either TAS (*n* = 26, 33%) or SRC C1–C2 (*n* = 52, 67%). In 11 cases, the SRC was extended to include C3. In total, 201 C1- and C2-polyaxial screws (16 of these with modified positioning through lamina due to anatomical reasons), and 52 transarticular screws were placed (Figure 1) (Table 2).

### 3.3. Short-Term Outcome and Adverse Events

Intraoperative complications were noted in 2 cases (2.6%), both cases of vertebral artery injury during fixation with SRC. One occurred during C1-screw insertion and was treated with endovascular coiling. The other was a minor bleed during C2-drilling, but where angiography showed an intact lumen of the vessel and no additional treatment was needed.

The postoperative CT-scan showed screw malposition in 5 cases (2 TAS and 3 SRC). One case of TAS had no attachment to C1 on one side and the other had both screws positioned laterally, breaching into the transverse foramen on both sides. The SRC-cases were 1 lateral C1-screw with no bone purchase, 1 medial C1-screw breaching into the spinal canal, and 1 case where both C1-screws were considered inadequately positioned, with the latter two cases requiring revision surgery. During the routine follow up period of 3 months, there was one additional case of surgical revision: a patient treated with SRC had a sudden debut of neck pain 1 month after surgery, where a CT-scan showed a C2 screw migration on one side and the patient was revised with an extended posterior fixation.

Long term follow-up showed 1 case of extended posterior fixation due to renewed minor cervical trauma one year after surgery (SRC at index surgery) were CT-scan showed resorption zones around the screws. There was also 1 conversion from TAS to SRC due to remaining pain after more than a year and with 1 of the screws fractured on CT. There was 1 case of tethered cord (discovered 6 years after surgery). An additional 2 transarticular screws were found fractured on the 3 months follow up CT, these did not require revision (Table 3).

### 3.4. Functional Outcome

The clinical follow-up time was 6.8 (1.0–15) years (excluding patients with death related to trauma < 1 month of surgery (*n* = 3)). Compared to each patients’ preoperative status, surgery was associated with significant decrease in the incidence of pain (cervicalgia), from 88% to 26% (*p* < 0.001). Complete pain relief was noted in 65% (*n* = 45), and partial pain relief in 19% (*n* = 13) of the total 69 cases with data available for comparison. Ten percent showed no pain relief and 5.8% (*n* = 4) had no pre-operative pain and remained unchanged. No patients developed pain after surgery. Motor deficit, sensory deficit, balance disorder and bladder dysfunction were not significantly decreased. Moreover, improved Frankel grade was seen in 3 (4.3%) patients, while 66 (90%) were unchanged and there were no cases of deterioration (*n* = 69 cases available for comparison) (Table 4 and Table 5).

## 4. Discussion

This study evaluated 78 consecutive cases of atlantoaxial instability treated with posterior C1–C2 fixation. The results are in accordance with previously published studies suggesting that non-navigated posterior C1–C2 fixation, with both TAS and SRC, is an effective treatment for cervical instability and pain [2,3,4,5,6,10,11,17,18,19,20,21,22,23,24,25,26,27,28,29,30,31,32]. Three patients (3.8%) underwent revision to improve construct stability, in 2 cases due to renewed trauma with extended posterior fixation (C0-C4 and C0-Th2) and in 1 case due to fractured TAS combined with neck pain, where the TAS-construct was removed in favor of SRC. Only 10% of patients had no pain relief at all. Previous studies have shown equivalent results concerning stability and surgical risks comparing the two surgical techniques [3,5,10,11,12,23].

In our series, median OR time was slightly longer for SRC (148 min (excluding constructs involving C3)) compared to TAS (139 min). However, studies differ in reporting total OR time or only surgical time making the comparison difficult [11]. Hitti et al. reported a significantly longer mean OR time of 198 min using an O-arm-based navigation compared to 157 min for non-navigated surgery. Nonetheless, navigated OR time decreases with experience and approaches values reported for non-navigated surgery [33]. Yang et al. reported a shorter, although not significant, OR time using iso-centric C-arm 3D navigation for TAS compared to fluoroscopy [34], indicating that user experience and surgical technique may impact surgical time more than the implementation of navigation per se.

The reported blood loss in our material was lower for TAS compared to SRC, which is in line with previous reports and is logical since TAS is a minimally invasive technique [11]. The difference can further be explained by damage to the venous plexuses during dissection for C1 screw placement. With an overall median estimated blood loss of 350 mL in our material, there is room for improvement. Reports have shown that use of navigation may reduce blood loss for both surgical techniques [33,34]. Arguably, this is explained by a less invasive approach and reduced manipulation in the proximity of the cervical venous plexuses.

A well-known and potentially dangerous complication of screw malplacement associated with both TAS and SRC is vertebral artery injuries [3,11,12,23]. In this study, there were 2 cases of vertebral artery injury corresponding to 2.6% of all patients treated and 1.0% of all polyaxial screws that were placed. Wright and Lauryssen surveyed 847 active members of the American Association of Neurological Surgeons/Congress of Neurological Surgeons to quantitate the risk of vertebral artery injury during C1–C2 transarticular screw placement. One hundred and one respondents (47.4%) had placed a total of 2492 C1–C2 transarticular screws in 1318 patients and the risk of VAI was 4.1% per patient, or 2.2% per screw inserted [22]. A recent study from 2019 with 127 screw-rod constructs showed vertebral artery canal breaches in 5.1% of the screws applied, and vertebral artery occlusion in 4 patients (3%) [17]. Both cases of vertebral artery injury in our material occurred during polyaxial screw insertion (SRC) and the incidence is similar to previous reports. Notably, no neurological complications were observed in these cases. A meta-analysis by Elliot et al. found that the incidence of malpositioned screws or vertebral artery injury was <0.5%. The authors reported that variations in the screw starting point impacted the accuracy of C1 screw insertion and that sacrifice of the C2 nerve root resulted in fewer malpositioned screws [35]. At our center, sacrifice of the C2 nerve root is actively avoided to reduce postoperative sensory loss. Despite this, the number of malplaced C1 screws was only 4 (4.1%). In this fluoroscopy-assisted study, a high level of accuracy (98.0% of polyaxial and 94.2% of the transarticular screws) was achieved, assessed on postoperative CT (Figure 1).

### 4.1. Future Perspectives and Strategies to Avoid VAI

One of the main arguments for implementation of surgical navigation in cervical spine surgery is to avoid potentially dangerous complications such as VAI during instrumentation. Although navigation does not eliminate this risk [36], various reports on navigational techniques and screw templates have shown improvements in screw accuracy compared to non-navigated free-hand surgery.

Alternative strategies to avoid VAI include probing technologies based on doppler, impedance or optical properties. Lofrese et al., described a doppler ultrasound technology for intermittent intraoperative monitoring of the vertebral artery. Doppler probing was performed during lateral dissection, stepwise drilling, and tapping, to avoid VAI in the intervention group. They argued that the technique represents a useful tool supporting fluoroscopy-assisted as well as navigated C1–C2 surgeries [37]. The Pediguard, an electronic conductivity device (SpineGuard, Paris, France) designed to assist in pedicle screw placement, has also been used to avoid VAI in fluoroscopy-assisted cadaveric procedures [38,39]. Diffuse Reflectance Spectroscopy is an optical technique which has been experimentally integrated into a surgical device to detect impending cortical breach during pedicle screw placement [40,41]. The continued evolution of percutaneous minimally invasive techniques for cervical fixation procedures will depend on navigation solutions as well as supportive technologies such as those mentioned above. The development and implementation of novel supportive technologies promises to increase the safety of all types of instrumented spinal surgery. However, technological solutions cannot be afforded in all countries. In the meantime, non-navigated C1–C2 fixation, in experienced hands, remains a valid option for atlantoaxial stabilization.

### 4.2. Limitations

This is a retrospective study with inherent limitations. The study cohort is quite heterogenous as it includes patients treated for both traumatic and degenerative disorders using two different surgical techniques. Although navigation may improve accuracy and reduce the complication rate, these effects will need to be verified in controlled studies. A randomized control trial comparing navigated and non-navigated C1–C2 screw fixation is needed to better evaluate differences and possible advantages of navigation.

## 5. Conclusions

C1–C2 posterior stabilization with both TAS and SRC, is a safe and effective treatment for atlantoaxial instability and provides pain relief in the vast majority of the cases. In this fluoroscopy-assisted study, a high screw placement accuracy was achieved with a low rate of intraoperative complications and few surgical revisions.

## Figures and Tables

**Table 1:** Baseline data.

Variable	Value (*n* = 78)
Age (years)	63 (16–83)
Male sex	36 (46%)
ASA-class	3 (1–4)
Prior C1–C2 surgery	4 (5.1%)
Dens screw fixation	3
C1–C2 softwire	1
*Clinical presentation*	
Motor deficit	11 (14%), (2 missing, 2.6%)
Sensory deficit	8 (10%), (3 missing, 3.8%)
Balance disorder	9 (12%), (5 missing, 9.5%)
Bladder dysfunction	3 (3.8%), (4 missing, 5.1%)
Pain	69 (88%), (3 missing, 3.8%)
*Frankel grade*	*n* = 76
A	0 (0%)
B	1 (1.3%)
C	5 (6.6%)
D	5 (6.6%)
E	65 (85.5%)
Pre-operative computed tomography angiography	43 (55%)
Pre-operative MRI performed	46 (59%)
Intramedullary high T2 signal intensity	9 (12%)
**Surgical Indication**	
Acute trauma	27 (35%)
Trauma > 1 week	23 (29%)
*Type of traumatic injury*	
Isolated C1-fracture	6
Isolated dens type 2-fracture	26
Isolated dens type 3-fracture	9
Ruptured transvers ligament, no fracture	1
C1-fracture + dens type 2-fracture	3
C1-fracture + dens type 3-fracture	2
Dens type 2-fracture + Hangman’s fracture	1
Dens type 3-fracture + Hangman’s fracture	2
Rheumatic instability	9 (12%)
Os odontoideum	3 (4%)
Pseudarthrosis (previous surgery)	2 (3%)
Other degenerative atlantoaxial disorders	14 (18%)

Data presented as median (range) or number (proportion). Abbreviations: ASA = American Society of Anesthesiologists.

**Table 2:** Treatment data.

Variable	Value (*n* = 78)
Time from diagnosis to surgery (days)	84 (0–3327) days
Acute Trauma	2 (0–7) days
Non-union	104 (14–851) days
Other elective surgery	300 (7–3327) days
*Surgical method*	
SRC C1–C2	41 (53%)
(Including laminectomy)	5
SRC C1–C3	11 (14%)
TAS	26 (33%)
(Including laminectomy)	2
Total number of polyaxial screws in C1 and C2	201
Total number of atlantoaxial transarticular screws	52
*Operative time*	150 (64–306) min
SRC C1–C2	148 (80–305) min
SRC C1–C3	174 (113–224) min
TAS	139 (64–306) min
*Intraoperative blood loss*	350 (25–2100) mL
SRC C1–C2	400 (50–2100) mL
SRC C1–C3	600 (100–1400) mL
TAS	150 (25–450) mL

Data presented as median (range) or number (proportion). Abbreviations: SRC = Screw-rod constructs, TAS = Transarticular screw.

**Table 3:** Treatment outcome.

Variable	Value (*n* = 78)
*Intraoperative complications*	2 (2.6%)
Vertebral artery injury (no intervention)	1
Vertebral artery injury (with postoperative coiling)	1
*Surgical revision (<3 months)*	3 (3.8%)
Screw revision (SRC)	2
Extended posterior fixation (SRC)	1
*Other adverse events*	10 (13%)
Pneumonia	5
Angioedema	1
Superficial surgical site infection	1
Bacteremia	1
Non-ST Elevation Myocardial Infarction	1
Cerebral fat-embolism	1
*Long-term follow up*	
Extended posterior fixation	1
TAS converted to SRC	1
Tethered spinal cord	1
*Radiological follow-up*	
Screw malposition on postoperative CT	7
Screw fracture	2
Construct instability	1

Data presented as mean (standard deviation) or number (proportion). Abbreviations: SRC = Screw-rod constructs, TAS = Transarticular screw.

**Table 4:** Functional outcome.

Variable	Value (*n* = 78)
Duration of follow-up (years)	6.8 (1.0–15) years *
Death during follow-up	16 (21%)
Time from surgery to death	895 (5–4078) days
Death due to cervical instability	0 (0%)
Motor deficit	7 (9%), (7 missing, 9.0%)
Sensory deficit	3 (3.8%), (8 missing, 10%)
Balance disorder	4 (5.1%), (10 missing, 13%)
Bladder dysfunction	2 (2.6%), (9 missing, 12%)
Pain	20 (26%), (8 missing, 10%)
*Change in pain*	
Data available	69
*Positive result*	58 (90%)
Complete pain relief	45 (65%)
Partial pain relief	13 (19%)
No preoperative pain and remained unchanged	4 (6%)
*Negative result*	7 (10%)
No pain relief	7 (10%)
New postoperative pain	0
*Frankel grade*	
Data available	70
A	0
B	0
C	1 (1.5%)
D	5 (7.1%)
E	64 (91.4%)
*Change in Frankel grade*	
Data available	69
Improved	3 (4%)
Unchanged	66 (96%)
Worsened	0 ^1^

^1,^* Excluding patients with death related to trauma < 1 month (*n* = 3). Data presented as median (range) or number (proportion).

**Table 5:** Functional outcome: statistics.

Variable	Pre-Operative (*n* = 78)	Post-Operative (*n* = 78)	*p*-Value
Motor deficit	11 (14%), (2 missing)	7 (9.0%), (7 missing)	1.000
Sensory deficit	8 (10%), (3 missing)	3 (3.8%), (8 missing)	0.248
Balance disorder	9 (12%), (5 missing)	4 (5.1%), (10 missing)	0.480
Bladder dysfunction	3 (3.8%), (4 missing)	2 (2.6%), (9 missing)	1.000
Pain	69 (88%), (3 missing)	20 (26%), (8 missing)	**<0.001**

Data presented as number (proportion). Bold text in the *p*-value column indicates a statistically significant correlation (*p* < 0.05).

## Data Availability

Data are available from the corresponding author upon reasonable request.

## Supplementary Materials

The following supporting information can be downloaded at: https://www.mdpi.com/article/10.3390/medicina58010114/s1, Table S1: Comparison between SRC and TAS.
