# Supplementary material for: Fluoroscopy-Assisted C1–C2 Posterior Fixation for Atlantoaxial Instability: A Single-Center Case Series of 78 Patients
Source: Medicina (Kaunas). 2022 Jan 12;58(1):114. doi: 10.3390/medicina58010114 (PMC8779556; doi:10.3390/medicina58010114)
Supplement: Supplementary file 1 [file medicina-58-00114-s001.zip › medicina-1512979-supplementary.pdf]

Supplementary Table S1. Comparison between SRC and TAS

Variable	SRC (Harms, *n* = 52)	TAS (Magrl, *n* = 26)	p-value
Male sex	30 (58%)	6 (23%)	**0.004**
Age (years)	63 (16 – 83)	62 (25 – 74)	0.984
Acute trauma	25 (48 %)	2 (7.7 %)	**< 0.001**
ASA class	3 (1 – 4)	2 (1 – 3)	0.144
C1 fracture	11 (21%)	0 (0%)	**0.012**
Dens type 2	26 (50%)	4 (15%)	**0.003**
Dens type 3	11 (21%)	2 (7.7%)	0.199
Ruptured transverse lig.	2 (3.8%)	1 (3.8%)	1.000
Hangman's fracture	3 (5.8%)	0 (0%)	0.547
Rheumatic instability	1 (1.9%)	8 (31%)	**< 0.001**
OR time (minutes)	155 (80 – 305)	143 (64 – 306)	0.412
Blood loss (ml)	500 (50 – 2100)	150 (25 – 500)	**< 0.001**
Screw revision	2 (3.8%)	0 (0%)	0.550
